# Using Direct Acyclic Graphs to Enhance Skeleton-Based Action Recognition with a Linear-Map Convolution Neural Network

**DOI:** 10.3390/s21093112

**Published:** 2021-04-29

**Authors:** Tan-Hsu Tan, Jin-Hao Hus, Shing-Hong Liu, Yung-Fa Huang, Munkhjargal Gochoo

**Affiliations:** 1 Department of Electrical Engineering, National Taipei University of Technology, Taipei 10608, Taiwan; thtan@ntut.edu.tw (T.-H.T.); t103310022@ntut.edu.tw (J.-H.H.); 2 Department of Computer Science and Information Engineering, Chaoyang University of Technology, Taichung 413310, Taiwan; 3 Department of Information and Communication Engineering, Chaoyang University of Technology, Taichung 413310, Taiwan; yfahuang@cyut.edu.tw; 4 Department of Computer Science & Software Engineering, College of Information Technology, United Arab Emirates University, Al Ain P.O. Box 15551, Abu Dhabi, United Arab Emirates; mgochoo@uaeu.ac.ae

**Keywords:** linear-map convolutional neural network, direct acyclic graph, action recognition, spatial feature, temporal feature

## Abstract

Research on the human activity recognition could be utilized for the monitoring of elderly people living alone to reduce the cost of home care. Video sensors can be easily deployed in the different zones of houses to achieve monitoring. The goal of this study is to employ a linear-map convolutional neural network (CNN) to perform action recognition with RGB videos. To reduce the amount of the training data, the posture information is represented by skeleton data extracted from the 300 frames of one film. The two-stream method was applied to increase the accuracy of recognition by using the spatial and motion features of skeleton sequences. The relations of adjacent skeletal joints were employed to build the direct acyclic graph (DAG) matrices, source matrix, and target matrix. Two features were transferred by DAG matrices and expanded as color texture images. The linear-map CNN had a two-dimensional linear map at the beginning of each layer to adjust the number of channels. A two-dimensional CNN was used to recognize the actions. We applied the RGB videos from the action recognition datasets of the NTU RGB+D database, which was established by the Rapid-Rich Object Search Lab, to execute model training and performance evaluation. The experimental results show that the obtained precision, recall, specificity, F1-score, and accuracy were 86.9%, 86.1%, 99.9%, 86.3%, and 99.5%, respectively, in the cross-subject source, and 94.8%, 94.7%, 99.9%, 94.7%, and 99.9%, respectively, in the cross-view source. An important contribution of this work is that by using the skeleton sequences to produce the spatial and motion features and the DAG matrix to enhance the relation of adjacent skeletal joints, the computation speed was faster than the traditional schemes that utilize single frame image convolution. Therefore, this work exhibits the practical potential of real-life action recognition.

## 1. Introduction

Recently, the lifespans of the world’s population are increasing, and society is gradually aging. According to the report of the United Nations [1], the number of elderly people (over 65) in the world in 2019 was 703 million, and this is estimated to double to 1.5 billion by 2050. From 1990 to 2019, the proportion of the global population over 65 years old increased from 6% to 9%, and the proportion of the elderly population is expected to further increase to 16% by 2050. In Taiwan, the report of the National Development Council indicated that the elderly population with age over 65 will exceed 20% of the national population at 2026 [2].

Taiwan will enter a super-aged society in 2026. This means that the labor manpower will gradually decrease in the future. Thus, the cost of home care for elders will significantly increase. In homecare, the monitoring of elderly people living alone is a major issue. The behaviors of their activities have a high relation with their physical and mental health [3,4]. Therefore, how to use artificial intelligence (AI) techniques to reduce the cost of home care is an important challenge.

The recognition of body activities has two major techniques. One is physical sensors, like accelerometers [5,6], gyroscopes [7], and strain gauges [8], which have the advantage that they can be worn on the body to monitor dangerous activities throughout the day and the disadvantage that few activities can be recognized. Therefore, the physical sensors are typically not used to identify the daily activities. Another technique is the charge-coupled device (CCD) camera [9,10], which has the advantage of being able to recognize many daily activities and the disadvantage that it can only be used to monitor the activities of people in a local area. Thus, it is suitable to be used in a home environment.

Many previous studies have used deep learning techniques to recognize daily activities, including two-stream convolutional neural networks (CNNs), long short-term memory networks (LSTMNs), and three-dimensional CNNs (3D CNNs). For the two-stream CNN, Karpathy et al. used context stream and fovea streams to train a CNN [11]. The two streams proposed by Simonyan et al. were spatial and temporal streams, which represent the static and dynamic frames of each action’s film [12]; however, the spending time for each action was different.

Thus, Wang et al. proposed a time segment network to normalize the spatial and temporal streams [13]. Jiang et al. used two streams as the input combined with CNN and LSTMN [14]. Ji et al. proposed 3D CNN to obtain the features of the spatial and temporal streams [15]. The two-stream methods using image and optical flow to represent the spatial and temporal streams had a better performance for recognizing activities compared with the one-stream methods. However, the weakness is that its doubled data amount requires more time to train the model.

Studies have used skeletal data as the common input feature for human action recognition [16,17,18], where the 3D skeletal data were typically obtained by use of the depth camera. In these studies, the number of recognized actions was less than 20 [17], and the skeletal data had to be processed to extract the features. Machine learning methods were used in these studies. The spatiotemporal information of skeleton sequences was exploited using recurrent neural networks (RNNs) [19,20].

Both the amount of data and recognized actions were less than in the video datasets [16,17,18,19,20]. An RNN tends to overemphasize the temporal information and ignore the spatial information, leading to low accuracy. However, the advantage of methods employing skeletal data is that these requires less training data and training time compared to those using image data. Hou et al. used a CNN to recognize actions with skeletal features [21]. Therefore, an effective method to encode the spatiotemporal information of a skeleton sequence into color texture images that could be recognized by a CNN is a relevant issue.

A directed acyclic graph (DAG) consists of a combination of nodes and edges. Each node points to another node by an edge. These directions do not become a circle graph that will end at the extremities. DAGs are usually used to represent causal relations amongst variables, and they are also used extensively to determine which variables need to be controlled for confounding in order to estimate causal effects [22]. The physical posture of people can be described by the positions of the skeletal joints. The adjacent joints have a causal relation when the body is moving. Thus, we can define the DAG of skeletal joints to explain the relations of physical skeletons.

This study aims to recognize the daily activities with films recorded by CCD cameras. To reduce the large amount of data for model training, we transferred body images to physical postures with an open system, AlphaPose [23]. The posture information is the skeleton sequences captured from the films of actions to build the spatial and motion features. These features all include both the spatial and temporal characteristics of actions. The relations of adjacent joints were used to build direct acyclic graph (DAG) matrices, the source matrix, and the target matrix.

These features are expanded by the DAG matrices as color texture images. The linear-map CNN has a two-dimensional linear map at the beginning of each layer to adjust the number of channels. Then, a two-dimensional CNN is used to recognize the actions. A structure with two streams was used to increase the accuracy of the action recognition. The datasets (NTU RGB+D) used in this study is an open source supported by Rapid-Rich Object Search Lab, National Technological University, Singapore [18].

In our work, a total of 49 actions, including daily actions, medical conditions, and mutual actions, were considered for action recognition. The total number of films was 46,452 for the cross-subject and cross-view sources. Of the cross-subject sources, 32,928 films were used for training, and 13,524 films were used for testing. Of the cross-view sources, 30,968 films were used for training, and 15,484 were used for testing. The experimental results show that the performance of our method was better than those in the previous studies.

## 2. Methods

**Figure 1:**
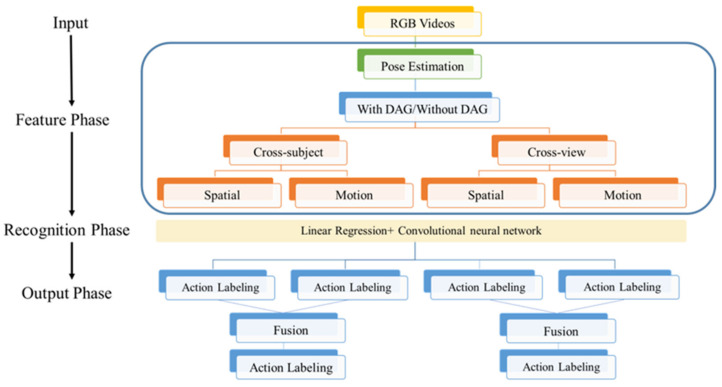
The flowchart of the action recognition in this study.

Figure 1 shows the flowchart of action recognition in this study, which has three phases. In the feature phase, RGB images were processed by AlphaPose [23] to obtain the coordinate values of the skeletal joints of the subject in an image as a vector. A film contained 300 images that were used to build a posture matrix as the feature. We defined the spatial features and motion features by the coordinate values of the skeletal joints for each film. Spatial features are the position information of skeletons and joints, and motion features are the optical-flow information of skeletons and joints.

Each feature was expanded into two features of source and target by DAGs. In the recognition phase, a 10-layer linear-map CNN was used to recognize the activities. The cross-subject and cross-view evaluations were used to test the performance of this linear-map CNN. In the output phase, the results of the spatial and motion features were fused to show the recognized actions.

### 2.1. NTU RGB+D Dataset

The datasets of action recognition supported by the Rapid-Rich Object Search Lab, National Technological University, Singapore [24] were used in the study. There were 56,880 files, including 60 action classes. Each file consists of RGB, depth, and skeleton data of human actions. All actions were recorded by three Kinect V2 cameras. The size of the RGB images was 1920 × 1080. There were 40 classes for daily actions, 9 classes for medical conditions, and 11 classes for mutual actions. Forty distinct subjects were invited for this data collection.

The physical activities of only a single person were recognized, and the sample size of 46,452, including the 49 physical activities, was used in this study. To ensure standard evaluations for all the reported results on the benchmark, two types of action classification evaluation (cross-subject evaluation, and cross-view evaluation) were used [24]. In the cross-subject evaluation, the sample sizes for training and testing sets were 40,320 and 16,560, respectively. In the cross-view evaluation, the sample sizes for training and testing sets were 37,920 and 18,960, respectively.

### 2.2. Spatial and Motion Features

The RGB images were processed by the AlphaPose [23] to obtain the coordinate values of skeleton joints of people in the image, and the format is shown in Equation (1).

(1) $$\mathrm{pose}=\left\{\left(x_{0},y_{0},c_{0},\cdots ,x_{M},y_{M},c_{M}\right)∥M=17\right\}$$

where *x_i_* and *y_i_* are the coordinate values of *i*th joint, *c_i_* is the confidence score, and *M* is the index of the joints. According to the coordinate values of the joints, the spatial and motion variables were defined, as shown in Table 1. *n* is the index of the frames. The spatial variables are the joint data (*v_i_*), and skeleton data (*s_i,j_*). The motion variables are the motion data of the joints and skeleton (*m_vi_* and *m_si_*). Thus, there are four features in this study, *F_v_*, *F_s_*, *F_mv_*, and *F_ms_*. Table 2 shows the indexes and definition of 18 joints and 17 edges in the body. The 17 edges, e*_i_*, are defined as the relations between two adjacent joints, *i-1*th and *i*th.

### 2.3. Directed Acyclic Graph

DAG was used to describe the relations of 18 joints. The nodes of DAG represent the joints, and the flows represent the edges. Each edge has a source joint and a target joint. Thus, two DAG matrices, the source matrix and target matrix, can be defined. If the *i*th joint is the source point of the *j*th edge, the element (*j*, *i*) of the source matrix is set as 1. Otherwise, the element (*j*, *i*) is set as 0.

**Figure 2:**
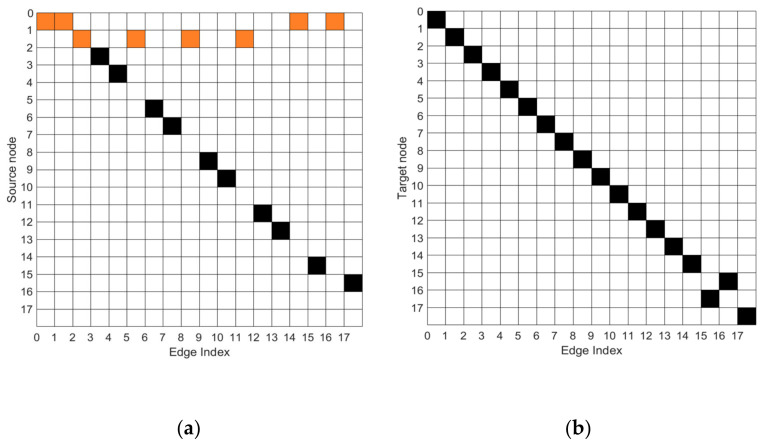
(**a**) Source matrix, (**b**) Target matrix. Color-none is 0, color-black is 1, and color-orange is 0.25.

The target matrix is set as the source matrix. Then, each row of the source and target matrices is normalized to avoid overvalues. To match the size of feature, e(0, 0) = 1 is defined as a virtual edge. The sizes of the source and target matrices are 18∗18. Figure 2a is the source matrix, S, color-none is 0, color-black is 1, and color-original is 0.25. Figure 2b is the target matrix, T.

### 2.4. Input Features

**Figure 3:**
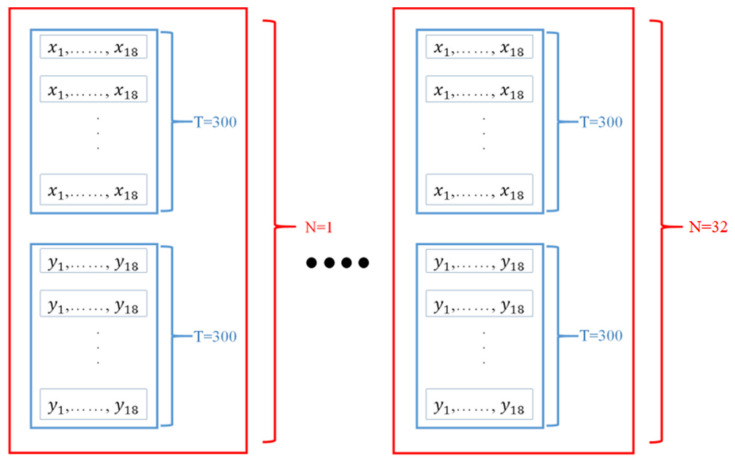
One feature built by the joint data, skeleton data, joint-motion data, or skeleton-motion data, with the size of 600∗18.

We used 300 frames for every film. The joint data built the joint feature (*F_v_*), the skeleton data built the skeleton feature (*F_s_*), the joint-motion data built the joint-motion feature (*F_mv_*), and the skeleton-motion data built the skeleton-motion feature (*F_ms_*). Figure 3 shows the contents of a feature with x and y values of a data. Thus, the information of the film was reduced to four 600*18 matrices for four features, *F_v_*, *F_s_*, *F_mv,_* and *F_ms_*. The *F_v_* was expanded by the DAG matrix into two features, *F_vin_* and *F_vout_*.

(2) $$F_{vin}=F_{v}\times S^{T}$$

(3) $$F_{vout}=F_{v}\times T^{T}$$

*F_s_* was expanded by the DAG matrix to *F_sin_* and *F_sout_*; *F_mv_* was expanded to *F_mvin_* and *F_mvout_*; and *F_ms_* was expanded to *F_msin_* and *F_msout_*. Table 3 shows the contents of the spatial feature (*F_spatial_*) and motion feature (*F_motion_*). *F_saptial_* is the combination of *F_spatial-joint_* and *F_spatial_skeleton_*. *F_motion_* is the combination of *F_motion-joint_* and *F_motion_skeleton_*. We used the two features (*F_spatial_* and *F_motion_*) to evaluate the performance of the linear-map CNN.

### 2.5. Linear-Map CNN

**Figure 4:**
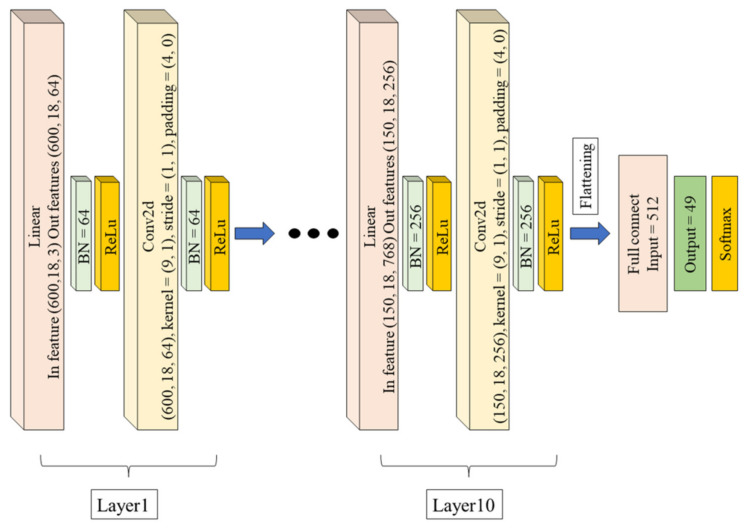
The structure of the 10-layer linear-map CNN.

Figure 4 shows the structure of a 10-layer linear-map CNN. The linear-map was used to adjust the number of channels at the beginning of each layer. Batch normalization (BN) can overcome the disappearance of the learning gradient and, thus, use a larger learning rate. In the CNN, the kernel size is a 9 × 1 matrix, the stride is (1,1), and the padding is (4,0). In the input feature, columns represent the different joints, and rows represent the time sequence of the actions. The relation of the adjacent joints was enhanced by the DAG matrix. Thus, the kernel of convolution is a 9 × 1 matrix. Table 4 shows the detailed information of the linear-map CNN. The output layer has 49 nodes representing the 49 action classes. The optimal method was momentum. The batch number is 32.

### 2.6. Statistical Analysis

According to our proposed method, a film is considered as true positive (TP) when the classification action is correctly identified; false positive (FP) when the classification action is incorrectly identified; true negative (TN) when the action classification is correctly rejected, and false-negative (FN) when the action classification is incorrectly rejected. Here, the performance of the proposed method was evaluated using these parameters,

(4) $$Precision\left(\%\right)=\frac{TP}{TP+FP}\times 100\%,$$

(5) $$Recall\left(\%\right)=\frac{TP}{TP+FN}\times 100\%,$$

(6) $$Specificity\left(\%\right)=\frac{TN}{TN+FP}\times 100\%,$$

(7) $$F_{1}score=\frac{2\times Precision\times Recall}{Precision+Recall}\times 100\%,$$

(8) $$Accuracy\left(\%\right)=\frac{TP+TN}{TP+TN+FP+FN}\times 100\%.$$

## 3. Results

In this study, the hardware employed was CPU Intel Core i7-8700 and GPU GeForce GTX1080. The operating system was Ubuntu 16.04LTS software, the development system was Anaconda 3 at python 3.7 version, the tool of deep learning was Pytorch 1.10, and the compiler was Jupyter Notebook. We evaluated the performance of DAG with the cross-subject and cross-view sources, and four features (*F_spatial_joint_*, *F_spatial-skeleton_*, *F_motion_joint_*, and *F_motion_skeleton_*). At last, we used the two-stream concept, class score fusion for *F_spatial_* and *F_motion_*, to evaluate the performance of the proposed method with cross-subject and cross-view sources.

Table 5 shows the results without the DAG transfer. The best feature is *F_spatial_* under the cross-subject source, which resulted in an accuracy and F1-score of 99.3% and 82.8%, respectively. There were 10 actions with recall rates below 70%: 0, 4, 9, 10, 11, 16, 28, 29, 43, and 48. The worst feature is *F_motion_* under the cross-subject source, its accuracy and F1-score are 99.2% and 79.6%, respectively. There are 11 actions with recall rates below 70%: 3, 10, 11, 16, 24, 28, 29, 31, 36, 43, and 45. Table 6 shows the results with the DAG transfer. The best feature was *F_spatial_* under the cross-view source; its accuracy and F1-score were 99.9% and 96.2%, respectively.

Only four actions, 10, 11, 28, and 29, had recall rates below 70%. The worst feature was *F_motion_* under the cross-subject source, which obtained an accuracy and F1-score of 99.1% and 79.1%, respectively. There were 10 actions with recall rates below 70%: 2, 10, 11, 16, 28, 29, 31, 43, 44, and 45. We found that the DAG transfer could significantly improve the recognition rate of different actions, not only for spatial features but also for motion features. Table 7 shows the results of class score fusion with and without DAG transfer. We found that the performance of DAG transfer used in the cross-view source was better than used in the cross-subject source, with an accuracy of 99.9% vs. 99.5% and F1-score of 94.7% vs. 86.3%. The recall rates for all 49 actions were not below 70%.

We used the two-dimensional joint and skeleton features to perform the training and testing of the linear-map CNN, which could reduce the running time more than those using two- or three-dimensional joint and skeleton images. Table 8 shows the training and testing time with and without DAG transfer. We found that the GPU could process about 30 frames/second (fps) in the training phase, and process about 125 fps in the testing phase. Although the DAG transfer required time to process, the delay time was about 30 min in the training phase. The maximum testing time was 141 s.

## 4. Discussion

In this study, we used DAG transfer and the two-stream method to improve the accuracy of action recognition. When the input features were transferred with the DAG matrices, the precision, recall, specificity, F1-score, and accuracy were improved by 1.2%, 1.1%, 0.1%, 1.3%, and 0.1%, respectively, for the cross-subject source, and were improved by 9.1%, 7.4%, 0.1%, 7.4%, and 0.5%, respectively, for the cross-view source. In the two-stream method, previous studies have typically used the spatial and temporal, or optical flow features to perform the active score fusion [11,12,13,14]. They also proved that the performance of two streams was better than one stream.

We used joint and skeleton sequences as the spatial motion features that had temporal characteristics. We utilized 300 frames to describe an action. Thus, the spatial feature of one action included the spatial and temporal characters. However, the motion relations of the joints and skeletons were different from one action to another. Therefore, we defined the motion variables, *mv_i_* and *ms_i_*, as shown in Table 1, to establish the motion features.

Our results also show that the precision, recall, specificity, F1-score, and accuracy of the two streams were improved by 0% vs. 6.3%, 0.1% vs. 7.4%, 0% vs. 0.2%, 0.1% vs. 7.1%, and 0.1% vs. 0.4%, over spatial and motion streams with the DAG transfer in the cross-subject source, and improved by 0.5% vs. 3.9%, 0.4% vs. 4.5%, 0% vs. 0%, 0.5% vs. 4.3%, and 0% vs. 0.2% in the cross-view source.

The comparison of our results with the previous studies under the recall rate is shown in Table 9. These studies all used cross-subject and cross-view sources from the NTU RGB+D database to recognize actions and also used three-dimensional characteristics of each posture as the input features [19,25,26,27,28,29,30,31]. Our method had the best recall rates of 86.1% and 94.7% in the cross-subject and cross-view sources.

**Figure 5:**
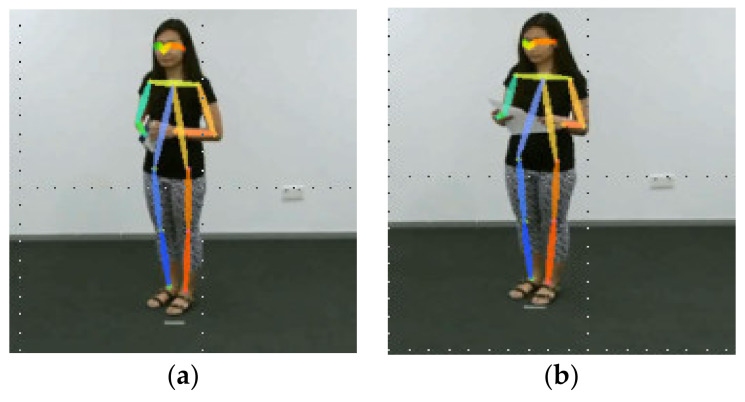

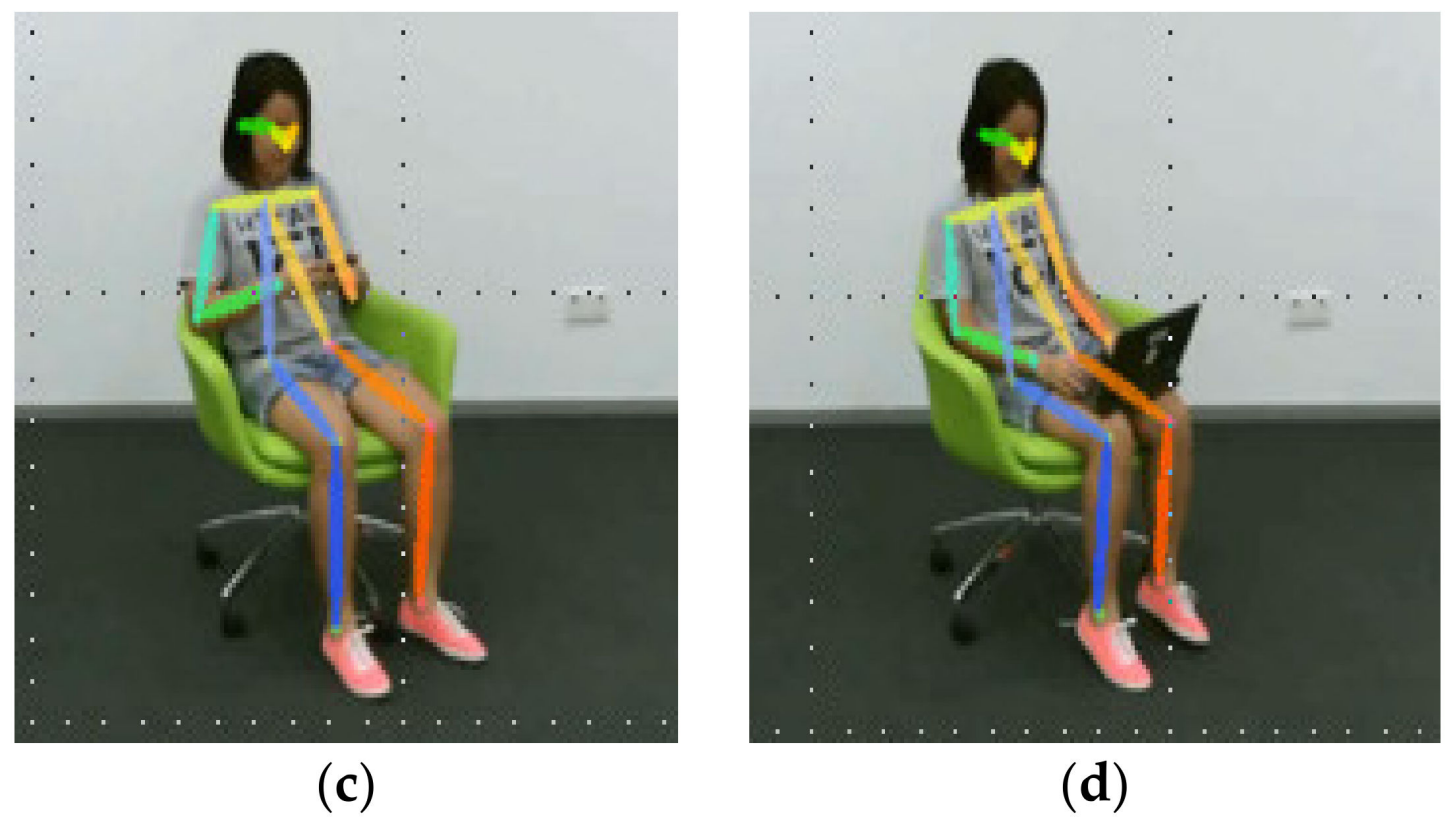
(**a**) the posture of reading, (**b**) the posture of writing, (**c**) the posture of making a phone call, (**d**) the posture of using the laptop.

We analyzed the actions with lower recall rates in the cross-subject and cross-view sources in Table 5, Table 6 and Table 7. The four actions that often had lower recall rates were A10 (reading), A11 (writing), A28 (phone call), and A29 (playing with laptop). Figure 5a is the posture of reading, and Figure 5b is the posture of writing. The subject is standing up, looking down, and holding something. The difference between the two images is only in the gestures of two hands. However, according to the description of the body posture in Table 2, only the right and left wrist joints are marked, which cannot show the gestures of two hands.

The postures of the subject making a phone call (in Figure 5c) and using the laptop (in Figure 5d)) had the same problem. The difference between the two images was also in the gestures of the two hands. These actions were difficult to recognize using spatial features, such as the movement trajectories of the arms, elbows, and wrists. Thus, the results of our method with the DAG transfer and two-stream method in Table 7 show that no action had a lower recall rate in the cross-view source.

## 5. Conclusions

The large scale of the collected data in the NTU RGB+D database enabled us to apply the posture-driven learning method for action recognition. The posture information represented by the skeleton data was obtained from the 300 frames of the film. The joint and skeleton sequences were used to build spatial and motion features that included the spatial and temporal characteristics of the actions. The relations of the adjacent skeletal joints were used to build the DAG matrices.

The spatial or motion features were expanded by DAG matrices as color texture images. The expanded features all indicated that the relations between adjacent joints were enhanced. Our method effectively reduced the amount of data for training the linear-map CNN, and its performance was superior to the previous schemes using deep learning methods. Notably, since the computation speed can reach around 125 fps in the testing phase with GPU, our scheme could be used to monitor the daily activities of elders in real time in home care applications.

## Figures and Tables

**Table 1:** The indexes of the joints and relations between every two adjacent joints at the 17 edges.

**Spatial Variables**	
**Joint Data**	**Skeleton Data**
$v_{i}\left(n\right)=\left(x_{i}\left(n\right),y_{i}\left(n\right)\right)$	$s_{i,j}\left(n\right)=\left(x_{i}\left(n\right)-x_{j}\left(n\right),y_{i}\left(n\right)-y_{j}\left(n\right)\right)$
**Motion Variables**	
**Joint-Motion Data**	**Skeleton-Motion Data**
$mv_{i}\left(n\right)=v_{i}\left(n+1\right)-v_{i}\left(n\right)$	$ms_{i}=s_{i,i+1}\left(n+1\right)-s_{i,i+1}\left(n\right)$

**Table 2:** The indexes and positions of the joints and edges for the skeletal data.

Index	Joint Position	Index	Joint Position	Indexe of Joints and Edges
0	Nose	9	Right knee	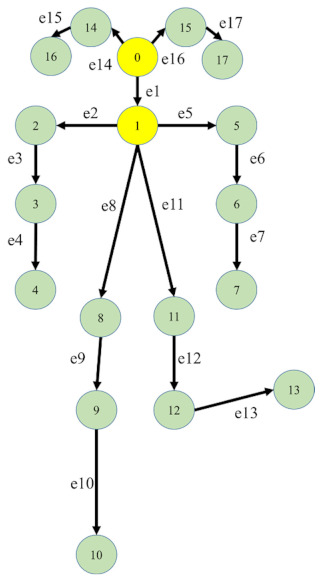
1	Neck	10	Right ankle	
2	Right shoulder	11	Left femur	
3	Right elbow	12	Left knee	
4	Right wrist	13	Left ankle	
5	Left shoulder	14	Right eye	
6	Left elbow	15	Left eye	
7	Left wrist	16	Right ear	
8	Right femur	17	Left ear	

**Table 3:** The channel contents of the input features.

*F_spatial_* (1200,18,3)	*F_spatial_joint_* (600,18,3)	(*F_v_*, *F_sin_*, *F_sout_*)
	*F_spatial_skeleton_* (600,18,3)	(*F_s_*, *F_vin_*, *F_vout_*)
*F_motion_* (1200,18,3)	*F_motion_joint_* (600,18,3)	(*F_mv_*, *F_msin_*, *F_msout_*)
	*F_motion_skeleton_* (600,18,3)	(*F_ms_*, *F_mvin_*, *F_mvout_*)

**Table 4:** The parameters of linear-map CNN in each layer.

	Linear	BN	AF	Conv2d	BN	AF
L1	In feature (600,18,3), Out features (600,18,64)	64	ReLu	(600,18,64), kernel = (9,1), stride = (1,1), padding = (4,0)	64	ReLu
L2	In feature (600,18,192), Out features (600,18,64)	64	ReLu	(600,18,64), kernel = (9,1), stride = (1,1), padding = (4,0)	64	ReLu
L3	In feature (600,18,192), Out features (600,18,64)	64	ReLu	(600,18,64), kernel = (9,1), stride = (1,1), padding = (4,0)	64	ReLu
L4	In feature (600,18,192), Out features (600,18,64)	64	ReLu	(600,18,64), kernel = (9,1), stride = (1,1), padding = (4,0)	64	ReLu
L5	In feature (600,18,192), Out features (600,18,128)	128	ReLu	(600,18,128), kernel = (9,1), stride = (2,1), padding = (4,0)	128	ReLu
L6	In feature (300,18,384), Out features (300,18,128)	128	ReLu	(300,18,128), kernel = (9,1), stride = (1,1), padding = (4,0)	128	ReLu
L7	In feature (300,18,384), Out features (300,18,128)	128	ReLu	(300,18,128), kernel = (9,1), stride = (1,1), padding = (4,0)	128	ReLu
L8	In feature (300,18,384), Out features (300,18,256)	256	ReLu	(300,18,256), kernel = (9,1), stride = (2,1), padding = (4,0)	256	ReLu
L9	In feature (150,18,768), Out features (150,18,256)	256	ReLu	(150,18,256), kernel = (9,1), stride = (1,1), padding = (4,0)	256	ReLu
L10	In feature (150,18,768), Out features (150,18,256)	256	ReLu	(150,18,256), kernel = (9,1), stride = (1,1), padding = (4,0)	256	ReLu
Flatten	Input 512 Output 49		Softmax			

**Table 5:** The results without the DAG transfer.

	Feature (Epochs)	Precision (100%)	Recall (100%)	Specificity (100%)	F1-Score (100%)	Accuracy (100%)	Actions (Recall < 70%)
Cross-subject	*F_spatial_* (70)	84.2%	82.8%	99.7%	82.8%	99.3%	0, 4, 9, 10, 11, 16, 28, 29, 43, 48
	*F_motion_* (65)	80.8%	79.7%	99.6%	79.6%	99.2%	3, 10, 11, 16, 24, 28, 29, 31, 36, 43, 45
Cross-view	*F_spatial_* (90)	86.4%	82.0%	99.7%	81.6%	99.3%	4, 9, 11, 16, 18, 28, 43, 44
	*F_motion_* (20)	82.9%	79.7%	99.7%	79.7%	99.2%	4, 9, 10, 11, 28, 29, 31, 32

**Table 6:** The results with the DAG transfer.

	Feature (Epochs)	Precision (100%)	Recall (100%)	Specificity (100%)	F1-Score (100%)	Accuracy (100%)	Actions (Recall < 70%)
Cross-subject	*F_spatial_* (65)	86.9%	86.0%	99.9%	86.2%	99.4%	10, 11, 28
	*F_motion_* (35)	80.6%	78.7%	99.7%	79.1%	99.1%	2, 10, 11, 16, 28, 29, 31, 43, 44, 45
Cross-view	*F_spatial_* (65)	94.3%	94.3%	99.9%	94.2%	99.9%	10, 11, 28, 29
	*F_motion_* (65)	90.9%	90.2%	99.9%	90.4%	99.7%	10, 11, 28, 29

**Table 7:** The results of class score fusion with and without DAG transfer.

		Precision (100%)	Recall (100%)	Specificity (100%)	F1-Score (100%)	Accuracy (100%)	Actions (Recall < 70%)
Cross-subject	Non GAD	85.7%	85.0%	99.8%	85.0%	99.4%	10, 11, 16, 28, 29, 31
	GAD	86.9%	86.1%	99.9%	86.3%	99.5%	10, 11, 28, 29
Cross-view	Non GAD	89.8%	87.3%	99.8%	87.3%	99.6%	5, 10, 12, 29
	GAD	94.8%	94.7%	99.9%	94.7%	99.9%	

**Table 8:** The training and testing time with and without DAG transfer.

Without DAG					
	Date Source	Feature	Epoch	Time: Day: Hr.: Min.:Sec.	Fps
Train	Cross-subject	Spatial	120	1:12:41:00	30
		Motion	92	1:04::04:00	30
Train	Cross-view	Spatial	120	1:11:39:00	30
		Motion	92	1:02:55:00	30
Test	Cross-subject	Spatial		0:00:02:01	125
		Motion		0:00:01:57	125
Test	Cross-view	Spatial		0:00:02:18	125
		Motion		0:00:02:19	125
**With DAG**					
Train	Cross-subject	Spatial	120	1:13:27:00	30
		Motion	92	1:04:29:00	30
	Cross-view	Spatial	120	1:11:05:00	30
		Motion	92	1:03:30:00	30
Test	Cross-subject	Spatial		0:00:02:03	125
		Motion		0:00:02:03	125
	Cross-view	Spatial		0:00:02:21	125
		Motion		0:00:02:19	125

**Table 9:** These studies all used cross-subject and cross-view sources in the NTU RGB+D database to recognize the actions, which also used the three-dimensional characteristics of each posture as the input features [19,25,26,27,28,29,30,31]. Our method had the best recall rates in the cross-subject and cross-view sources at 86.1% and 94.7%.

Methods	Recall Rate (%)	
	Cross-Subject	Cross-View
Lie Group [25]	50.1	52.8
HBRNN [19]	59.1	64.0
Deep RNN [26]	59.29	64.09
Deep LSTM [26]	60.7	67.3
Part-aware LSTM [26]	62.9	70.3
ST-LSTM + Trust Gate [27]	69.2	77.7
Two-stream RNN [28]	71.3	79.5
Clips + CNN + MTLN [29]	79.6	84.8
ST-GCN [30]	81.5	88.3
SR-TSL [31]	84.8	92.4
Proposed DAG + linear-map CNN	86.1	94.7
